# Comparison of the Transcriptomic and Epigenetic Profiles of Gonadal Primordial Germ Cells of White Leghorn and Green-Legged Partridgelike Chicken Embryos

**DOI:** 10.3390/genes12071090

**Published:** 2021-07-19

**Authors:** Aleksandra Dunislawska, Maria Siwek, Katarzyna Stadnicka, Marek Bednarczyk

**Affiliations:** Department of Animal Biotechnology and Genetics, UTP University of Science and Technology, 85-796 Bydgoszcz, Poland; aleksandra.dunislawska@utp.edu.pl (A.D.); siwek@utp.edu.pl (M.S.); katarzyna.stadnicka@utp.edu.pl (K.S.)

**Keywords:** gene expression, methylation, primordial germ cells, expression microarray

## Abstract

The Green-legged Partridgelike fowl is a native, dual-purpose Polish chicken. The White Leghorn has been intensively selected for several decades to mainly improve reproductive traits. Primordial germ cells (PGCs) represent the germline stem cells in chickens and are the only cells that can transfer the information stored in the genetic material from generation to generation. The aim of the study was to carry out a transcriptomic and an epigenetic comparison of the White Leghorn and Green-legged Partridgelike gonadal PGCs (gPGCs) at three developmental stages: days 4.5, 8, and 12 of the embryonic development. RNA and DNA were isolated from collected gPGCs. The RNA was further subjected to microarray analysis. An epigenetic analysis was performed based on the global methylation analysis and qMSP method for the particular silenced genes demonstrated in transcriptomic analysis. Statistically significant differences between the gPGCs from both breeds were detected on the day 8 of embryonic development. Global methylation analysis showed significant changes at the methylation level in the White Leghorn gPGCs on day 8 of embryonic development. The results suggest faster development of Green-legged Partridgelike embryos as compared to White Leghorn embryos. Changes in the levels of gene expression during embryonic development are determined by genetic and environmental factors, and this variability is influenced by breed and gender.

## 1. Introduction

Intensive genetic selection of chicken for the economically important production traits, which has been carried out for almost a century, has resulted in the considerable differences in the mechanisms of growth and development and, as a consequence, in the metabolism and reproduction of avians [1]. These differences might be identified at the early stages of avian embryogenesis [2,3,4]. Domestic chicken is also an important model for research in experimental embryology [5] focusing on the molecular basis of development and cell–cell interactions [6]. It is also used for studies in the fields of genomics [7], experimental medicine [8], and epigenetics [9].

Primordial germ cells (PGCs) represent the germline stem cells in chicken, which can be identified at an early stage X of Hamburger–Hamilton (HH). Further differentiation of these cells depends on the formation of the embryo [10]. Initially, they are attached to the posterior side of the hypoblast, but as growth progresses, they move and finally reach the anterior part of the blastodisc, the so-called germinal crescent. When blood vessels are formed, PGCs, called circulating PGCs (cPGCs), pass into the embryonic bloodstream, through which they enter the genital ridges. After settling into the future gonadal region, gonadal PGCs (gPGCs) proliferate and begin to differentiate into male and female reproductive cells [11]. After a period of mitotic proliferation, gPGCs undergo meiosis and differentiate into functional gametes—either ova or sperms. Post-mating, the fertilization of ova and sperm initiates embryogenesis. Subsequently, new PGCs begin to be produced in the embryo. Thus, PGCs are the only cells that can transfer the information stored in the genetic material from generation to generation. They are potentially of a great importance in understanding the epigenetic regulatory mechanisms in vertebrates. However, due to limited cell numbers, the epigenetic regulation of gene expression during the PGC determination and cell migration in vivo has been rarely studied [12].

In our previous study on gPGCs isolated from an intensively selected breed, White Leghorn (WL), we investigated the dynamics of the transcriptome during chicken embryo development [13]. We concluded that the changes in the gene expression depend on the time at which the PGCs were isolated during embryonic development and the highest number of genes was regulated between days 8 and 12 of an incubation. However, the study lacked information about the effect of sex and breed of the embryo on the molecular level in chicken PGCs. In the current study, we compared the leading breed in egg production—WL chicken—with a common native dual-purpose chicken (Green-legged Partridgelike—GP) in an identical experimental set-up of transcriptomic analysis and performed global and gene-specific methylation analysis in both the breeds of chicken considering the effect of gender. It is an innovative examination of the molecular changes occurring at the level of embryonic development related to gene expression.

This line of research was chosen based on an analysis of the WL transcriptome, which showed that intensive gene expression silencing also occurs. We assume that this process may be related to the DNA methylation. The impact of the DNA methylation on host genome depends on the gene region in which it occurs. DNA methylation of regulatory elements suppresses gene expression. Hence, DNA methylation of gene-deficient regions is considered crucial for the maintenance of chromosome structure and integrity. Methylation of CpG dinucleotides might be inherited through cell division and transmitted to the next generation by germ cells. DNA hypermethylation of these dinucleotides causes silencing of gene expression, whereas DNA hypomethylation of these is related to gene expression [14].

In this study, we hypothesize that changes at the level of gene expression during embryonic development are determined by genetic factors, and the time variation in their occurrence is determined by breed and gender. The study is a follow-up transcriptomic and epigenetic analysis of gPGCs in two chicken lines with different genetic backgrounds.

## 2. Materials and Methods

**Figure 1:**
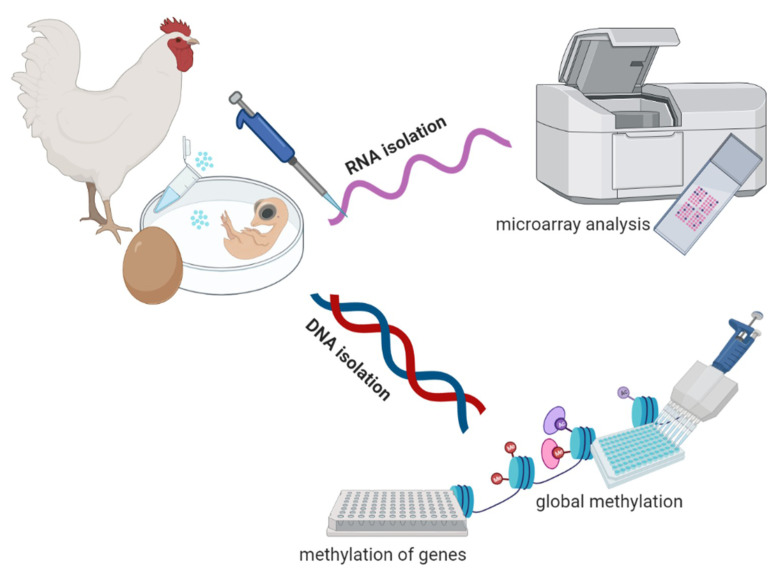
Experimental set-up (created with BioRender.com).

Experimental set-up is presented in Figure 1.

### 2.1. Gonadal PGC Isolation

Fertilized eggs of GP were incubated at 37.8 °C for 4.5, 8, and 12 days to obtain embryos of suitable developmental stage. Gonadal PGCs (*n* = 6/each group) were collected based on the method described by Nakajima et al. [15]. Gonads were obtained from the embryos on day 4.5, 8, and 12 of incubation (stage: 26, 34, and 38, respectively, according to Hamburger and Hamilton, 1951) [16]. The gonads were cultured for up to 90 min at 37.8 °C in phosphate-buffered saline without Ca^2+^ and Mg^2+^ (PBS [–]) to obtain live gonadal germ cells. The discharged, viable PGCs were identified by the morphological criteria and qPCR identification [13]. The PGCs have a typically spherical shape with a nucleus as the pale rounded structure. PGCs have numerous lipid droplets in their cytoplasm.

### 2.2. RNA and DNA Isolation

The PGCs were collected in a lysis buffer, and RNA was isolated by using a commercial kit for single-cell application according to the manufacturer’s protocol (GenElute Single Cell RNA Purification Kit, Sigma-Aldrich, St. Louis, MO, USA). The quality and quantity of the RNA were estimated with a 2100 Bioanalyzer instrument using the Agilent RNA 6000 Nano Kit (Agilent Technologies, Santa Clara, CA, USA). The RNA integrity number was assessed during the analysis. RNA of only high quality and purity was subjected to further molecular analysis.

For the methylation analysis, DNA was isolated using a commercial kit for the isolation of gDNA from materials that contained its trace amounts (Sherlock AX, A&A Biotechnology, Gdynia, Poland). The DNA was assessed using a 2% agarose gel and a Nanodrop2000 spectrophotometer (Thermo-Fisher Scientific, Waltham, MA, USA). PGC-related markers were examined to show that the isolated nucleic acid was derived from PGCs, as described by Dunislawska et al. [13].

### 2.3. Sex Determination of Embryos

Sexing of embryos for methylation analysis was performed by PCR. The primer sequences and reaction conditions for detecting W chromosome-specific repeating sequences and control PCR to amplify the chicken 18S ribosomal gene were chosen based on Clinton et al. [17]. W chromosome sequence was chosen to amplify a 415 bp product (primer sequences—F: CCCAAATATAACACGCTTCACT; R: GAAATGAATTATTTTCTGGCGAC) and ribosomal gene sequence was selected to amplify a 256 bp product (primer sequences—F: AGCTCTTTCTCGATTCCGTG; R: GGGTAGACACAAGCTGAGCC). The reaction was performed in the presence of Color Taq DNA polymerase (EURx, Gdansk, Poland). After amplification, the reaction product was separated on a 2% agarose gel and visualized in a gBOX instrument (fluorescence gel imaging, Syngene, Frederick, MD, USA) following staining with SimplySafe dye (EURx, Gdansk, Poland). Females were detected based on the presence of a band of 415 bp, whereas a band of 256 bp was detected in the control reaction.

### 2.4. Transcriptome Analysis

Microarray analysis was carried out without gender division (samples of randomly selected individuals) with 100 ng RNA using SurePrint G3 Custom GE 8 × 60 k microarrays for *Gallus* (*n* = 4/each group). Single-color microarrays were chosen because they allow a comparison of each sample with others and provide an immediate reference at any time. For this purpose, three slides were used with four matrices on each. The analysis within a time point was performed in four independent biological replicates. The microarray preparation procedure was performed following the manufacturer’s protocol for one-color microarray-based gene expression analysis (Agilent Technologies, Santa Clara, CA, USA), which includes the following stages: (1) sample preparation—template RNA with a spike-in, (2) cDNA synthesis, (3) cRNA synthesis and amplification, (4) cRNA purification, (5) preparation of the hybridization sample, (6) 17 h of hybridization at 65 °C, (7) microarray wash, (8) scan in Agilent Microarray Scanner System—SureScan (Agilent G3 GX1 color protocol), and (9) extraction of data using Agilent Feature Extraction Software.

After scanning and feature extraction, the obtained data were analyzed by GeneSpring GX software, version 14.9 (Agilent Technologies, Santa Clara, CA, USA). The data were normalized and subjected to quality control in the software. The following criteria were used to analyze the obtained list of genes: statistical significance (*p* value) higher than 0.05 and cut-off value greater than 2.0 indicating upregulated genes and lower than—2.0 indicating downregulated genes (cut-off of fold change value > 2 or <−2). Statistical analysis was performed using one-way analysis of variance. The selected time points were also compared with each other and between genotypes (GP vs. WL) as described [13]. The Agilent microarray was validated in Dunislawska et al. for WL [13]. For qualitative analysis, a heat map was used to present the general direction of changes in expression. GeneSpring software was also used for the qualitative assessment to assign genes to the main terms of gene ontology (GO).

### 2.5. Global Methylation Analysis

DNA for global methylation analysis was intended for analysis taking into account the division into sex of individuals after prior PCR identification and prepared using a commercial kit for methylated DNA quantification (MDQ1, Imprint Methylated DNA Quantification Kit, Sigma-Aldrich) according to the manufacturer’s protocol. DNA isolated from gPGCs (*n* = 5 for each time point and each sex) was diluted in a binding solution to a final concentration of 150 ng/µL. Then, it was used for estimating the level of methylated DNA based on enzyme-linked immunosorbent assay on 96-well plates. Positive (methylated) and blank controls were analyzed together with the DNA samples. The absorbance was measured at 450 nm by using the Multiscan (Thermo-Fisher Scientific, Waltham, MA, USA) microplate reader and read by SkanIt software 6.0.2. (Thermo-Fisher Scientific, Waltham, MA, USA). For each time point (4.5, 8, and 12 days), five samples per group (male/female), each derived from a different individual, were analyzed. The absorbance measurements were taken in two technical repetitions, each time using the same amount of DNA. The two measurements were averaged, and the mean value was used for the further analysis. Global DNA methylation levels were expressed as percentages relative to the methylated control and were calculated using the following formula: $\frac{A_{450}S-A_{450}B}{A_{450}MC-A_{450}B}\times 100\%$, where $A_{450}S$ is the average absorbance of the sample, $A_{450}B$ is the average absorbance of the blank, and $A_{450}MC$ is the average absorbance of the methylated control. Statistical analysis was carried out by SAS Enterprise Guide 8.2 (SAS Institute Inc., Cary, NC, USA). The quantitative values were first analyzed for normality using the Shapiro–Wilk test. Obtained values failed normal distribution assumption; therefore, the Kruskal–Wallis test was used (the influence of sex and the influence of developmental day; *p* < 0.05).

### 2.6. Gene-Specific Methylation Analysis

The isolated DNA was also subjected to the methylation analysis using the real-time quantitative methylation-specific polymerase chain reaction (qMSP-PCR) as described by Dunislawska et al. [9].

Based on the microarray data (presented in the current publication and our previous manuscript [13]), genes that have been silenced and whose functions were assumed to be important for embryonic development were selected for qMSP.

The conversion was carried out using the EpiJet Bisulfite Conversion Kit (Thermo-Fisher Scientific/Fermentas, Vilnius, Lithuania) according to the manufacturer’s instructions. qPCR was performed for the selected genes. For each gene, two primer pairs were designed—specific for methylated and nonmethylated DNA—using the MethPrimer tool according to Dunislawska et al. [9]. Primers for qMSP were complementary to the gene promoter region and were designed using the following criteria: island size > 100, GC% > 50.0, and obs./exp > 0.60. DNA oligonucleotides were synthesized by Sigma-Aldrich (St. Louis, MO, USA) (sequences presented in Table 1). The qPCR analysis was performed in the LightCycler 480 (Roche Diagnostics, Risch-Rothreuz, Switzerland) thermal cycler. The reaction mixture contained the Maxima SYBR Green qPCR Master Mix intercalating dye (Thermo-Fisher Scientific/Fermentas, Vilnius, Lithuania). The optimized melting temperature was 58 °C. The relative level of DNA methylation (%) was calculated from the melting curves (red fluorescence level) for each DNA sequence according to the formula described by Dunislawska et al. [9]. The quantitative values were first analyzed for normality using the Shapiro–Wilk test. Obtained values failed normal distribution assumption; therefore, the Kruskal–Wallis test was used (the influence of sex, influence of genotype, and the influence of embryonic developmental day; *p* < 0.05).

## 3. Results

### 3.1. Transcriptome Analysis

**Figure 2:**
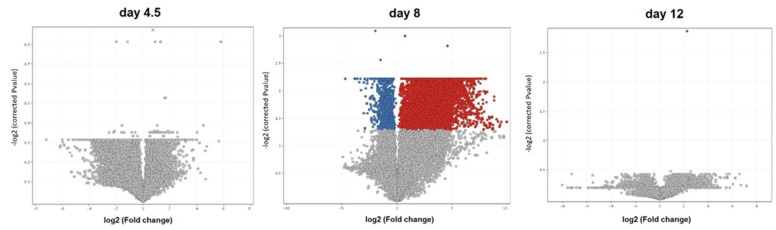
Volcano plot of genes expressed at the three time points of embryonic development of Green-legged Partridge-like (GP) chicken: 4.5, 8, and 12 days (red dots: upregulated genes; blue dots: downregulated genes statistically significant; gray dots: not statistically significant).

Comparative analysis showed changes in the expression (cut-off of fold change > 2 or <−2) in the GP only on day 8 of embryonic development. However, these differences between various time points of the GO embryo development were not statistically significant. The Volcano plot of the gene expression profiles at the studied time points are shown in Figure 2. The complete list of genes obtained in the microarray analysis is presented in Table S1 in supplementary file.

**Figure 3:**
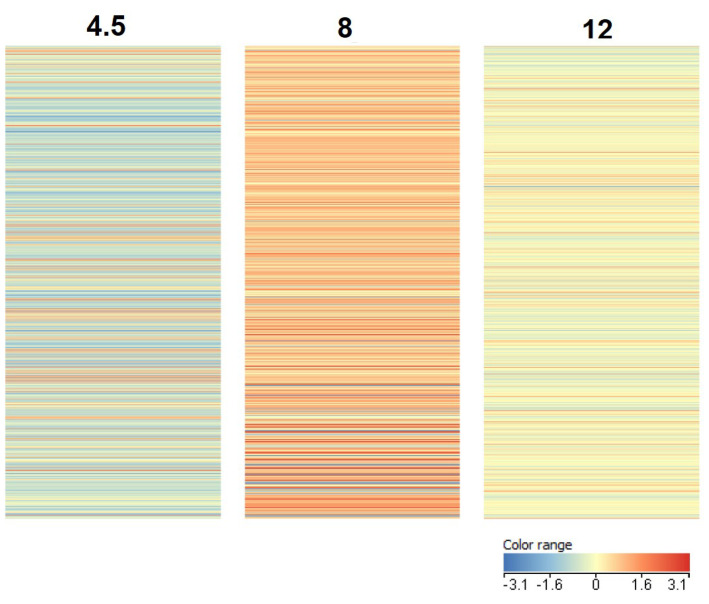
Heat maps of genes expressed at three time points of embryonic development of GP chicken: 4.5, 8, and 12 days.

The numerical analysis of the gene expression levels data obtained from the microarray at the three time points is presented by heat maps in Figure 3.

**Figure 4:**
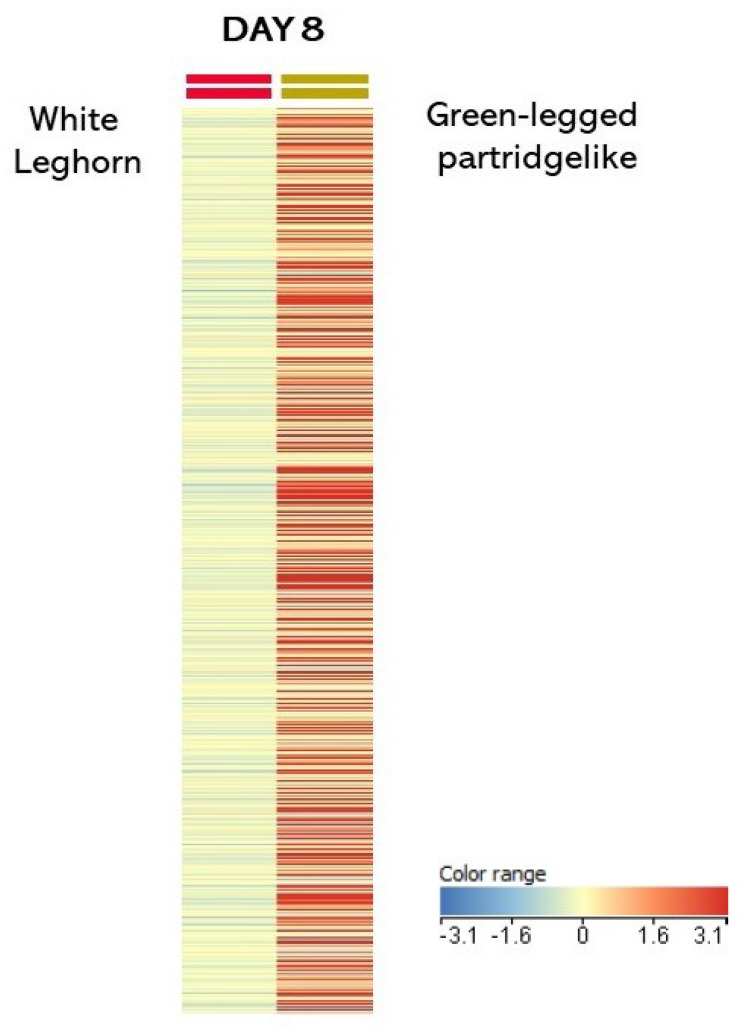
Heat maps of statistically significant differences in gene expression between two genotypes (comparison of GP to White Leghorn (WL)) on day 8 of embryonic development.

Statistically significant differences (*p* < 0.05) between PGCs from both breeds, GP and WL (data available in [13]) were detected on day 8 of embryonic development. Strong regulation of the gene expression (cut-off >2 or <−2) was demonstrated in the PGCs of GP as compared to those of WL. Differences in the global expression on day 8 of embryo development are presented in Figure 4.

Genes significantly differentiating GP and WL were found to be mostly involved in the pathways related to, e.g., the regulation of biological and cellular processes, developmental processes, anatomical structure development, and multicellular organism development and organ development. Table S2 in supplementary file shows a list of pathways in which regulated genes are involved and the number of genes involved categorized by GeneSpring GX software. In addition, Table S3 provides a list of genes that have been included in the reported GO terms.

### 3.2. Global Methylation Analysis

**Figure 5:**
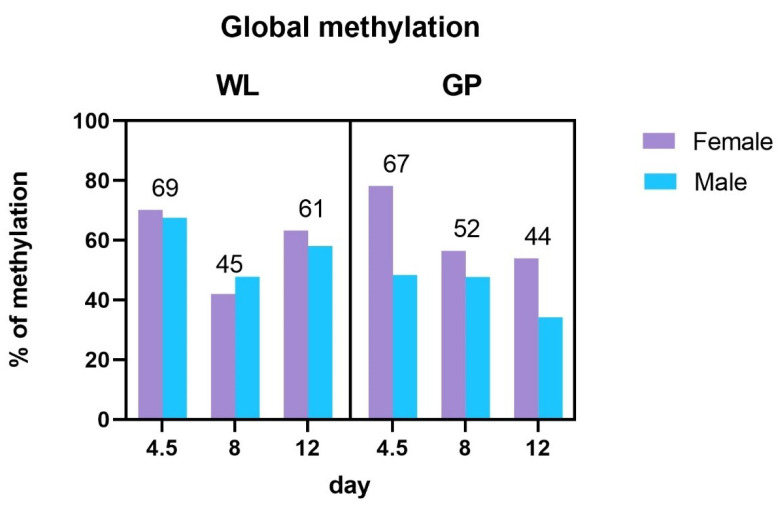
Global methylation levels (%) in three stages of embryonic development (days 4.5, 8, and 12) of two chicken breeds: WL and GP and two sexes (male and female).

There were no significant differences, but there were numerical differences between individual days and gender in the methylation analysis. In the case of the GP, the level of global methylation decreased with the following days of the embryonic development, which is especially noticeable in females. In WL, a decrease in methylation was observed on day 8, with a further increase on day 12. The results of global methylation analysis in gPGCs are presented in Figure 5.

### 3.3. Methylation of Silenced Genes

The selected genes for methylation analysis based on microarray data with statistically significant changes in the level of their expression are presented in Table 2.

**Figure 6:**
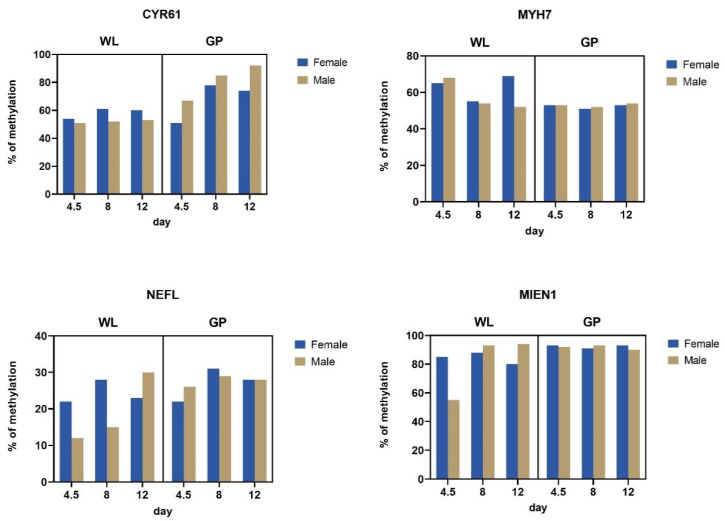
DNA methylation of the *CYR61, MYH7, NEFL*, and *MIEN1* genes in gonadal PGCs of WL and GP in three stages of embryonic development (days 4.5, 8, and 12) and categorized by sex. *X*-axis: days of embryonic development on which PGCs are collected; *Y*-axis: percentage of gene methylation.

There are statistically significant changes in the *CYR61* gene methylation observed between genotypes (WL vs. GP) (*p* < 0.05) and between genders in WL (*p* < 0.01). *CYR61* analyzed in the GP breed showed only a numerical (not statistical) increase in the methylation, especially in males. Statistically significant changes (*p* < 0.05) were also observed in the *MIEN1* gene in WL between the different days of embryonic development. The overall changes in gene methylation levels are shown in Figure 6. These results are consistent with the gene expression levels. The greatest negative regulation was observed for the expression of the *CYR61* gene both for the genotype and for the embryonic development day. In the case of methylation of the *MIEN1* gene, a significant decrease in expression on day 12 in WL is consistent with an increase in methylation.

## 4. Discussion

In this study, we aimed to verify the hypothesis that changes at the level of gene expression during embryonic development may be nongenetic and are determined by the breed and gender. We made such a hypothesis on the basis of our previous analyses, where it was shown that the day of incubation is important for the transcriptomic profile between individual embryonic stages in WL. On the basis of the obtained results, we decided to verify not only whether such a relationship is also observed in other breeds but also whether silencing the expression of individual genes may be related to the level of their methylation.

Two very different chicken breeds were subjected to this study. One of them is a GP, an old native, dual-purpose Polish chicken, which became recognized as a breed at the end of the nineteenth century. These chickens can adapt well to extreme environmental conditions and hence are considered to be more robust and disease-resistant than other breeds. Currently, the GP chicken breed is protected from extinction by in situ preservation. GP chickens have been kept in a flock without subjecting them to any selection process for more than 60 generations [18]. Our previous studies suggested that a GP can be an excellent model for research, for example, as a good source of PGCs [19] and hence has been extensively used for biotechnological manipulations [20,21].

The WL breed, on the other hand, has been intensively selected for several decades mainly to improve reproductive traits and has been used widely to create highly productive egg-laying hybrids for commercial and industrial operations. Literature reports suggest different numbers of cPGCs and gPGCs in both genotypes.

Kuwana et al. indicated a large variation in the PGC concentration even within the same breed [22]. The results of Szczerba et al. [19] showed that the number of gPGCs was probably influenced by the stage of embryonic development. In the case of inbred lines or endangered breeds, the number of total PGCs seems to be lower compared to commercial chicken breeds [23,24]. This lower number of PGCs seems likely to be correlated with the lower productivity of the local chicken. This is in line with the report of Zhao et al. [25] that the number of chicken PGCs correlated with the number of eggs. Several studies of the past have indicated that the consequences of intensive genetic selection of chicken for economically important production traits, which has been carried out for almost a century, can be detected in the early stages of avian embryogenesis [2,4,26].

### 4.1. Transcriptome Analysis

Differences in gene expression were detected on day 8 of embryonic development. This is a very specific time point of the development of an embryo when the female PGCs in the germinal epithelium are more numerous than in the earlier stages. At this stage, the PGCs undergo a rapid mitotic cell division and gonadal sex differentiation in the ovarian cortex [27]. Moreover, day 8 is marked by a rapid growth of external tissues in the chicken embryo [28]. The results of transcriptomic analysis obtained from the present and previous studies [13] suggest a faster development of the GP embryos compared to the WL embryos.

### 4.2. Global Methylation Analysis

Research shows that during embryogenesis, right after zygote formation, DNA demethylation takes place (the reverse process of methylation, in which a methyl group is removed from the molecule), and a new methylation profile begins to be established de novo from the blastocyst stage [29]. Gryzinska et al. showed that methylation is age related. An increase in the level of global DNA methylation in Polbar embryos was demonstrated during days 6–18 of embryonic development [30]. These authors also proved that global methylation levels decrease in individuals in the postembryonic phase. In our study, this relationship was confirmed in WL breed. The literature shows that the level of methylation is species and tissue specific [9,31], whereas the pattern of this process is determined during embryogenesis.

In our study, global methylation analysis confirmed that day 8 is crucial and that DNA methylation may be breed dependent. The results of the analysis obtained for both WL and GP are in line with Jang et al.’s study [14]. These authors performed DNA methylation chip analysis of PGCs for both genders and reported that DNA methylation is evenly distributed among male and female PGCs.

In our previous studies, we also found differences in global methylation levels between two different in ovo-stimulated chicken genotypes in the early stage of embryonic development. The analyses were performed on two genotypes of different origins and selection history. The difference observed in the analyses suggested that selection history and genetic diversity play a key role in the tissue-specific methylation process [32].

### 4.3. Gene-Specific Methylation Analysis

Analysis of individual gene methylation between WL and GP clearly shows breed-related changes in the gene profile. In WL, numerically greater differences in the levels of gene methylation were observed between the different days of embryonic development. Interestingly, in the transcriptome analysis in GP, no significant differences in gene expression were observed between different days, which may also translate into the lack of differences between days in the context of methylation.

The *CYR61* (cysteine-rich angiogenic inducer 61) gene is involved in the regulation of cell signaling and cell adhesion and in the developmental processes of the heart and skeleton. So far, the expression of this gene has been noted only in somatic cells [33], and its role in the development of gonads has not been reported. Our previous research has shown that this gene is silenced in the metabolic and immune tissues of adult chickens after stimulation of the intestinal microbiota during embryonic development [34]. However, it was observed that this silencing was not related to its methylation in the liver, spleen, or cecum tonsils in adult GP and broiler chicken [32]. Thus, silencing of gene expression in these cases may be related to RNA interference, and not DNA methylation. The present study showed numerically significant changes in the methylation levels of the *CYR61* gene of PGCs in GP while also considering the influence of gender. This result may suggest that these changes are closely related to the previously discussed mechanism of methylation decrease during embryonic development. It also may have a biological basis, given the role of this gene in the development of the heart. In the development of chicken embryo, the heart is the first organ to function, and it starts to develop in the HH2 stage [35].

The *MYH7* (myosin heavy-chain 7) gene encodes the β heavy-chain subunit of cardiac myosin. In our study, downregulation of the *MYH7* expression in WL was observed in the analyzed days of embryonic development. Interestingly, we observed positive regulation of its expression in GP on the eighth day of development and a decrease in its methylation in GP, regardless of the gender. *MYH7* has been described primarily in the context of hypertrophic cardiomyopathy in humans. However, chicken *MYH7* has been shown to have sequence similarity and evolutionary association with mutant human *MYH7.* Karunanithi et al. have pointed out the anatomical similarities between the heart of chicken and humans. These authors highlighted that MYH7 is one of the proteins responsible for heart contractions, and, therefore, abnormalities in the gene encoding this protein can lead to excessive thickening of the myocardium [36]. It is necessary to conduct further analyses in this direction, because in the conducted experiment no phenotypic analyses in this area were performed.

The *NEFL* (neurofilament light) gene showed slightly upregulated expression in GP on day 8 in the present study, thus showing no significant differences in methylation. In WL, its expression was clearly negative whereas methylation differed between days—especially in terms of sex (a significant increase in expression in the following days in males). This result suggests that the expression-silencing mechanism may be closely related to gene methylation. The significant function of this gene has been described as a typical neuronal marker of brain development based on the analysis of chicken embryos by high-throughput sequencing [37].

The *MIEN1* (Migration and invasion enhancer 1) gene is mainly involved in the regulation of apoptotic processes and cell migration. In WL, this gene shows a strong negative regulation during embryonic development between days 8 and 12, with a simultaneous increase in methylation from day 8. The level of its methylation in GP was found to be high for 4.5 days. Our previous studies showed that at the 14th stage of HH (i.e., about day 2) of embryonic development of GP, the peak total number of cPGCs and the maximum concentration were observed [19]. There was a decrease in concentration at a later stage, suggesting that cPGCs may be gradually trapped by capillary networks.

Our hypothesis that the GP embryo develops faster than the WL is supported by the statistically significant increase in methylation in WL observed between 4.5 and 8 days, while indicating a high level of methylation in GP from 4.5 days. In this case, methylation may be an important mechanism for silencing the expression of the genes that are responsible for cell migration.

## 5. Conclusions

PGCs allow a proper cell programming at the epigenetic level. Our research is the first step towards a comparative analysis of epigenetic mechanisms and their impact on gene expression silencing in two different genotypes of chickens. Based on our research, we can conclude that the transcriptomic profile differs according to the breed and stage of embryonic development. The analyses also confirmed that changes in the level of gene expression during embryonic development of WL and GP may be epigenetic and dependent on methylation. These changes are also determined by the sex and breed of the animal.

## Figures and Tables

**Table 1:** Sequences of the primers designed for qMSP reaction by using the MethPrimer tool.

Gene (NCBI No.)		Primer Sequences	GC%	Amplicon Size	Reference
*CYR61* (429089)	M	F: TTTGGTTTTAGTGTTTAAAGACGT R: TTATATTTACCTTCAAAAAAACGTA	58.33 44.00	150	[9]
	U	F: TTTTGGTTTTAGTGTTTAAAGATGT R: TATTTATATTTACCTTCAAAAAAACATA	56.00 42.86	154	
*MYH7* (395350)	M	F: AGGGTTTTGTTTCGTGTTTTATTC R: CTCCCCCATCTCTATAATAACGAT	70.83 62.50	100	This study
	U	F: GGGTTTTGTTTTGTGTTTTATTTGT R: CCTCCCCCATCTCTATAATAACAAT	76.00 64.00	100	
*NEFL* (419528)	M	F: TTTTTTGTATTCGGTGGATAGTTTC R: TAAAATCCTACAACTAAACCCGCT	68.00 70.83	104	This study
	U	F: TTTTTGTATTTGGTGGATAGTTTTG R: TTAAAATCCTACAACTAAACCCACT	68.00 68.00	104	
*MIEN1* (100858225)	M	F: GGGGTAGTTGAGAGTTATACGT R:TACAAAATAATACAAAAAAAACGAC	68.18 68.00	125	This study
	U	F: TGTGGGGTAGTTGAGAGTTATATGT R: TACAAAATAATACAAAAAAAACAAC	64.00 68.00	128	

M: specific for methylated DNA; U: specific for unmethylated DNA.

**Table 2:** Statistically significant gene expression changes detected on the microarray (GeneSpring software) in particular points of embryonic development of two chicken breeds—WL and GP.

Breed	Day	*CYR61*	*MYH7*	*NEFL*	*MIEN1*
WL	8 vs. 4.5	–3.26	–2.37	nd	nd
	12 vs. 4.5	–2.34	–3.91	–4.19	–2.52
GP	8 vs. 4.5	–1.78	1.30	0.37	nd

WL: White Leghorn; GP: Green-legged Partridgelike; nd: no statistically significant data.

## Data Availability

New data were created, analyzed, and presented in this study. Data sharing is not applicable to this article.

## Supplementary Materials

The following are available online at https://www.mdpi.com/article/10.3390/genes12071090/s1, Table S1: List of genes whose differentiation was demonstrated on the expression microarray by comparing GP and WL on day 8 of embryonic development; Table S2: Biological pathways (GO terms) involving genes whose different expression was observed on day 8 of embryonic development between GP and WL; Table S3: List of genes that have been classified into GO terms by the GeneSpring software.
